# Depression, anxiety, and stress levels during the COVID-19 pandemic: A longitudinal study among Indonesian psychologists

**DOI:** 10.1371/journal.pone.0315584

**Published:** 2025-10-17

**Authors:** Fitri Ariyanti Abidin, Ahmad Gimmy Prathama, Efi Fitriana, Anggi Mayangsari, Rahmi Salsabila Putri Syam, Sophia Amira Latifa Hakim, Joeri K. Tijdink

**Affiliations:** 1 Department of General, Developmental, and Educational Psychology, Faculty of Psychology, Universitas Padjadjaran, Padjadjaran, Indonesia; 2 Center for Relationship, Family Life and Parenting Studies, Faculty of Psychology, Universitas Padjadjaran, Padjadjaran, Sumedang, Indonesia; 3 Center for Innovation and Psychological Research, Faculty of Psychology, Universitas Padjadjaran, Sumedang, Indonesia; 4 Jakarta University National, Jakarta, Indonesia; 5 Bachelor of Psychology Study Program, Faculty of Psychology, Universitas Padjadjaran, Sumedang, Indonesia; 6 Department of Ethics, Law and Humanities, Amsterdam UMC, location VUmc, Amsterdam, the Netherlands; National Institutes of Health, University of the Philippines Manila / De La Salle University, PHILIPPINES

## Abstract

During the COVID-19 pandemic, while clinical psychologists played a crucial role in supporting mental health, their well-being was overlooked compared to other healthcare professionals. Therefore, this study aimed to examine the trend levels of depressive symptoms, anxiety, and stress among psychologists during the pandemic and explore how these levels are related to demographic characteristics, personality traits, and self-compassion. One hundred and ten psychologists who provided online counseling during the pandemic participated in all three-time points of the study conducted from January to October 2021 in the midst of the pandemic. The validated Indonesian versions of the DASS-21, Big Five Personality, and Self-Compassion Scale were surveyed. The results indicated that despite no significant longitudinal changes in stress, anxiety, and depression levels (p > 0.05) over nine months, the prevalence of moderate-to-severe depressive symptoms, anxiety, and stress was noted in 10.9%−14.5%; 22.7%−30.9%; and 11.8%−14.5%; respectively. In terms of protective and risk factors, being married, older age, higher openness to experience, higher extraversion, and higher conscientiousness emerged as potential protective factors against mental health issues. Additionally, self-compassion was linked to depression, anxiety, and stress at corresponding time points; however, its impact diminished over time. Meanwhile, stress emerged as a significant predictor of both depression and anxiety. These findings demonstrate that the mental health of psychologists during the pandemic was not severely affected over time. Compared with other populations, the levels of depressive symptoms, anxiety, and stress are lower. The results may indicate that Indonesian psychologists are able to cope with very stressful situations, like a global pandemic. Future studies should focus on protective factors.

## Introduction

The COVID-19 pandemic, initially regarded as an acute stressor, has gradually evolved into a chronic stressor with diverse mental health impacts, including an increased prevalence of psychopathology [1]. This trend has placed a considerable burden on mental health professionals worldwide. The government issued lockdown policies in April 2020 requiring all family members to stay at home for an indefinite period, resulting in disruptions to daily routines, strained family relationships, and heightened uncertainties [2–7].

Numerous studies have examined the psychological effects of the COVID-19 pandemic on various segments of society. For instance, prior research has reported elevated anxiety, depression, and heightened sensitivity to social risks among the general population [4]. Within Indonesia, studies have revealed that stress resulting from the pandemic has led to excessive fear and anxiety, depression, difficulty concentrating, stress, and psychosomatic disorders [2,8]. Additional research has indicated that fear of impending doom, excessive worrying, becoming easily agitated, and difficulty relaxing are among the most common anxiety symptoms experienced by respondents. Likewise, the most frequently cited depression symptoms include sleep disturbances, low self-confidence, fatigue, and loss of interest [8]. Previous studies have also reported that the perception of an economic crisis resulting from the pandemic has caused psychological stress among Indonesian mothers [9].

The magnitude of the changes brought about by the current crisis underscores the crucial role of psychology in our collective response. Psychologists have actively engaged in various facets, encompassing policy work, community support, healthcare professional assistance, and public outreach. Furthermore, they have contributed to scientific progress through the execution of rigorous research endeavors during the pandemic [10]. The actions undertaken by psychologists in response to the pandemic predominantly revolved around public education and aiding healthcare professionals in managing the outbreak. Psychologists have implemented behavior change strategies to foster knowledge and commitment to healthy behaviors, including medication adherence, screening practices, self-management/self-care, and vaccination acceptance [11].

At the same time, psychologists themselves were not immune to the pandemic’s impact. Even under normal circumstances, personal experiences of mental health problems were common among clinical psychologists [12], with many reporting occasional stress [13]. During COVID-19, the increasing demand for psychological services [14] added a burden to the already potentially stressful condition psychologists experience concerning their own and family health. Due to the lockdown policy, they had to adapt to the changes in the workflow, such as mastering online counselling [15]. Although extensive research has examined the mental health of healthcare professionals during the pandemic (e.g., [16,17]), the focus has primarily been on physicians and nurses. Far fewer studies have considered the psychological well-being of mental healthcare professionals, such as psychologists. This group deserves special attention because they face the dual challenge of addressing clients’ heightened psychological distress while managing their own stress, anxiety, and uncertainty. Unlike many other health professionals, psychologists often work without strong institutional support, face limited access to protective resources, and are under added pressure to transition to digital practice [18,19].

In Indonesia, these challenges were particularly acute: the pandemic triggered a surge in demand for mental health services, yet the psychologist-to-population ratio remains low, and professional infrastructure is less developed compared to high-income countries [20–24]. This context makes Indonesian psychologists especially vulnerable, as they serve as the primary providers of psychological care in a resource-constrained setting. However, limited information is available on their mental health status during the COVID-19 pandemic and on the factors that may influence their well-being. Beyond occupational context, individual differences also shape psychological outcomes during crises. Research on the general population has found that some demographic factors, such as gender [25,26], age [27–29], and marital status [30] are associated with psychological distress during the pandemic. Personality traits, particularly high levels of neuroticism traits, have also been identified as significant risk factors for depressive symptoms and psychiatric symptomatology, while conscientiousness and agreeableness served as potential protective factors for psychological distress [26,27,29,31,32]. Personality is defined as a dynamic organization, inside the person, of psychophysical systems that create the person’s characteristic patterns of behavior, thoughts, and feelings [33]. The most used model of personality traits is the Big Five Personality. It consists of Extraversion, Agreeableness, Conscientiousness, Neuroticism, and Openness to Experiences. Each Big Five represents a broad set of related behavioral characteristics [34]. In addition, several studies have documented the protective role of self-compassion as a psychological factor in reducing psychological distress [25,30,35,36]. Self-compassion refers to how we relate to ourselves in instances of perceived failure, inadequacy, or personal suffering. It can be organized into three broad domains: how people emotionally respond to suffering (with kindness or judgment), how they cognitively understand their predicament (as part of the human experience or as isolating), and how they pay attention to suffering (in a mindful or overly identified manner) [37]. Individuals with greater self-compassion are more likely to adopt resilient and adaptive coping strategies, thereby mitigating distress.

Given this background, the objectives of this study were twofold. First, descriptively, to examine the levels of depression, anxiety, and stress among Indonesian psychologists during the COVID-19 pandemic across two measurement points, and to explore whether these scores changed or remained stable over time. As limited prior evidence exists on psychologists’ mental health in Indonesia, no specific directional hypotheses were made regarding these changes. Second, hypothesis-driven, to test whether individual characteristics, including personality traits and self-compassion, predicted depression, anxiety, and stress levels over time.

## Materials and methods

### Preregistration

We preregistered our study on the Open Science Framework. The preregistration details can be accessed at https://osf.io/vzf7s. We made some adjustments to the study design after registration. We initially planned to measure self-compassion only at the first time point. However, in the actual study, we measured self-compassion at the data collection’s first and second time points.

### Study design

**Fig 1:**
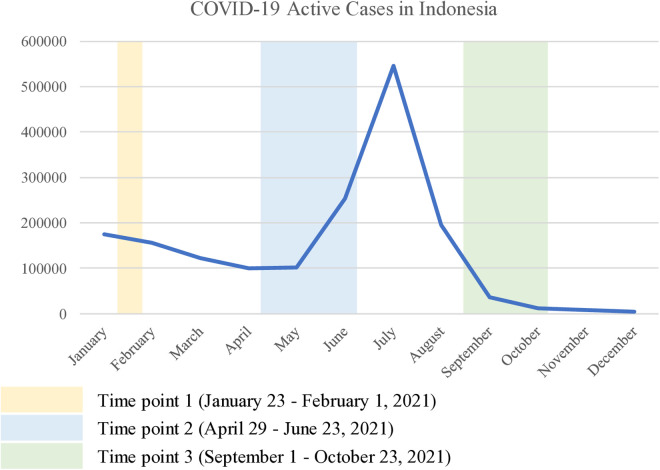
The National Epidemic Trend of Indonesia’s 2021 Coronavirus Disease (COVID-19) Outbreak from January 21 to December 21, 2021.

A longitudinal approach was employed to measure the psychological distress of psychologists every three months throughout the year 2021. The first survey was conducted from January 23 to February 1, 2021, the second from April 29 to June 23, 2021, and the third from September 1 to October 23, 2021. The first and second surveys were conducted during low infection rates in Indonesia, while the third survey was conducted after the number of infections peaked in 2021 (see Fig 1).

As written in the preregistration, we planned to collect data at three time points: January, May, and September 2021, to capture participants’ psychological states across different phases of the COVID-19 pandemic. The timing aligned with key developments in Indonesia’s public health policies. Time Point 1 (January) coincided with the early national vaccine rollout and moderate social restrictions (Pemberlakuan Pembatasan Kegiatan Masyarakat or PPKM); [38]. Time Point 2 (May–June) was conducted just before the sharp July surge, following increased mobility during the Eid holiday and amid regionally adjusted restrictions [39]. Time Point 3 (September–October) took place during the post-peak recovery phase, when PPKM Level 4 restrictions were being relaxed and vaccination coverage had expanded [40]. The longer data collection periods in Time Points 2 and 3 were necessary due to participants’ increased workload and reduced availability during the pandemic peak and recovery.

### Participants

Using G*Power analysis with an effect size of 0.15 [41], an α level of 5%, a power of 95%, and three predictor variables, the required minimum sample size was determined to be 119 participants. Given the challenges faced by participants during the COVID-19 pandemic and the attrition rates observed in longitudinal studies on COVID-19 (e.g., [42,43]), we sought to mitigate potential data loss by recruiting approximately three times the minimum required sample size. The final complete dataset (n = 110) closely approximated the expected sample size based on the GPower calculation. The participants are clinical psychologists who provided online counseling sessions during the COVID-19 pandemic. In the initial recruitment phase, an e-flyer containing information about eligibility criteria for participation, a direct link to the informed consent, and the survey was distributed through three different methods. Firstly, the Indonesian team posted the e-flyer on their personal social media, which was connected to a circle of psychologists. Secondly, the researcher invites potential participants by sharing the information directly with the closed psychologist WhatsApp groups. Thirdly, the researcher asked the leader of a regional psychologist association nationwide to share the e-flyer with their members. Those interested could access the online survey link after giving their approval through written informed consent. During the second stage of data collection, we distributed the survey link via email and WhatsApp to the participants who had consented. Follow-up messages were sent to those who had not completed the survey. Participants who did not respond to the third invitation to complete the survey were considered as attrition. The same procedure was followed during the third time point of data collection.

### Procedures

The Ethical Committee of Universitas Padjadjaran approved the research plan (Number 01/UN6.KEP/EC/2021). Participants were provided with detailed information about the study’s purpose, procedures, and potential risks and benefits. They were informed that participation was entirely voluntary and that they could withdraw from the survey at any time without consequences. Participants were assured of the confidentiality of their responses and the secure handling of their data. Participants provided their consent by clicking the “Agree” button. The participants completed a series of online questionnaires requiring approximately 10–20 minutes. The participants willing to participate in the subsequent time point were asked to provide their contact information. We utilized this information to contact them for participation in the next time point. An e-voucher valued at approximately $3 was randomly given to 50 participants at each time point as an incentive.

### Measurements

Demographic characteristics were measured using age, gender, marital status, and working experience questions.

To measure psychological distress, we utilized the Depression, Anxiety, and Stress Scale (DASS-21) [44]. The DASS-21 is comprised of three dimensions: depression, anxiety, and stress, each of which is measured by seven items, resulting in a total of 21 items. Participants were asked to rate each item using a 4-point Likert scale, ranging from 0 (Does not suit me at all, or never) to 3 (Perfectly suits me, or often). Sample items from the questionnaire include “I couldn’t seem to experience any positive feeling at all” (Depression), “I was aware of dryness of my mouth” (Anxiety), and “I found it hard to wind down” (Stress). The Indonesian version of the questionnaire has been validated [45]. The prevalence and severity of depression, anxiety, and stress symptoms were assessed following the recommendations of Lovibond and Lovibond [46]. Scores for each factor were calculated by summing up participant responses and then doubling the sum to obtain a final score. We classified participants into mild, moderate, severe, and extremely severe conditions of depression, anxiety, and stress based on the total score within each subdomain as follows: Depression scores were classified as: normal (0–9), mild (10–13), moderate (14–20), severe (21–27), and extremely severe (27+); Anxiety score was classified as: normal (0–7), mild (8–9), moderate (10–14), severe (15–19), and extremely severe (20+); Stress score was classified as: normal (0–14), mild (15–18), moderate (19–25), severe (26–33), and extremely severe (34+). In the present sample, the internal consistency (Cronbach’s alpha) of the DASS-21 at Time 1 was.86 for depression,.79 for anxiety, and.86 for stress, indicating acceptable to good reliability. The DASS-21 was administered at three time points, and the intraclass correlation coefficients (ICC) across time points were.629 for depression,.601 for anxiety, and.664 for stress, reflecting moderate temporal stability.

We employed the Indonesian version of the Big Five Inventory (BFI) [47] to measure personality. The Big Five Inventory (BFI) comprises 44 short-phrase items that measure five scales, i.e., Extraversion (E; 8 items), Agreeableness (A; 9 items), Conscientiousness (C; 9 items), Neuroticism (N; 8 items), and Openness (O; 10 items). The reliability in the current sample achieved satisfactory reliability scores for each dimension: Extraversion (.78), Agreeableness (.74), Conscientiousness (.78), Neuroticism (.84), and Openness (.71). Participants indicated their level of agreement or disagreement on a Likert scale from 1 (strongly disagree) to 5 (strongly agree). The Sample items included: “Is outgoing, sociable” (Extraversion), “Is helpful and unselfish with others” (Agreeableness), “Is a reliable worker” (Conscientiousness), “Is depressed, blue” (Neuroticism), and “Is curious about many different things” (Openness). The BFI was administered in the first point measurement.

Self-compassion was assessed using the Self-Compassion Scale [48], a 26-item questionnaire that measures six components of compassion: self-kindness (5 items, e.g., “I try to be understanding and patient towards those aspects of my personality I don’t like”), self-judgment (5 items, e.g., “When I see aspects of myself that I don’t like, I get down on myself”), common humanity (4 items, e.g., “When I feel inadequate in some way, I try to remind myself that feelings of inadequacy are shared by most people”), isolation (4 items, e.g., “When I fail at something that’s important to me, I tend to feel alone in my failure”), mindfulness (4 items, e.g., “When something upsets me, I try to keep my emotions in balance”), and over-identification (4 items, e.g., “When something upsets me, I get carried away with my feelings”). Participants used a Likert scale ranging from 1 (almost never) to 5 (almost always) to rate their responses. The Indonesian version of the questionnaire, which has been validated, was used [49]. The reliability in the present samples for each component ranged from 0.75 −.83. The Self-Compassion Scale was administered in the first two data collection time points.

### Data analysis

This study analyzed demographic data by presenting numbers and percentages, while descriptive statistics such as means and standard deviations were used. Given that the data did not follow a normal distribution, we used the Friedman Test to analyze the different scores of depression, anxiety, and stress between the three time points, with a predetermined significance level of 5%. The normality test shows that the data did not meet the normality. Kolmogorov-Smirnov test: Depression T1 Z = .225, p = .000; Anxiety T1 Z = .184, p = .000; Stress T1 Z = .161, p = .000; Depression T2 Z = .211, p = .000; Anxiety T2 Z = .200, p = .000; Stress T2 Z = .121, p = .000; Depression T3 Z = .204, p = .000; Anxiety T3 Z = .188, p = .000; Stress T3 Z = .131, p = .000.

To evaluate the relationship between demographic characteristics, personality traits, and self-compassion with depression, anxiety, and stress, a 4-step hierarchical linear regression analysis was conducted with T3 depression, anxiety, and stress as dependent variables. Gender, age, marital status, and working experience were entered as control variables in Step 1; extraversion, agreeableness, conscientiousness, neuroticism, and openness were entered in Step 2; self-compassion, depression, anxiety, and stress at T1 were entered in Step 3; self-compassion, depression, anxiety and stress at T2 was entered in step 4. We used raw (unstandardized) scale scores for all predictors in the regression analyses, as reported in Table 1. This approach follows previous studies that employed similar methods (e.g., [50]).

**Table 1:** Characteristics of Consented Eligible Participants.

Category	Timepoint 1 (n = 406)	Timepoint 1 Loss to follow up (n = 234)	Timepoint 2 (n = 172)	Timepoint 2 Lost to follow up (n = 62)	Timepoint 3 (n = 110)
**Age**	Range = 21–70 M (SD) = 38.03 (9.54)	Range = 23–63 M (SD) = 38.52 (9.42)	Range = 21–70 M (SD) = 37.36 (9.68)	Range = 26–69 M (SD) = 37.15 (9.58)	Range = 21–70 M (SD) = 37.48 (9.78)
**Working experience**	Range = 1–37 M (SD) = 8.26 (7.44)	Range = 1–33 M (SD) = 8.69 (7.73)	Range = 1–37 M (SD) = 7.67 (7.00)	Range = 1–30 M (SD) = 8.08 (7.24)	Range = 1–37 M (SD) = 7.44 (6.89)
**Gender, n (%)**					
** Female**	365 (89.9)	206 (88.0)	159 (92.4)	55 (88.7)	104 (94.5)
** Male**	39 (9.6)	26 (11.1)	13 (7.6)	7 (11.3)	6 (5.5)
** Missing**	2 (.5)	2 (.9)			
**Marital Status, n (%)**					
** Married**	291 (71.7)	171 (73.1)	120 (69.8)	44 (71.0)	76 (69.1)
** Non-Married**	114 (28.1)	62 (26.5)	52 (30.2)	18 (29.0)	34 (30.9)
** Missing**	1 (.2)	1 (.4)			
** **Depression	4.16 (5.70)		5.05 (6.32)		5.36 (5.98)
** **Anxiety	6.14 (6.31)		5.79 (5.92)		6.84 (6.84)
** **Stress	9.45 (7.92)		9.73 (8.21)		9.80 (8.28)

## Result

**Fig 2:**
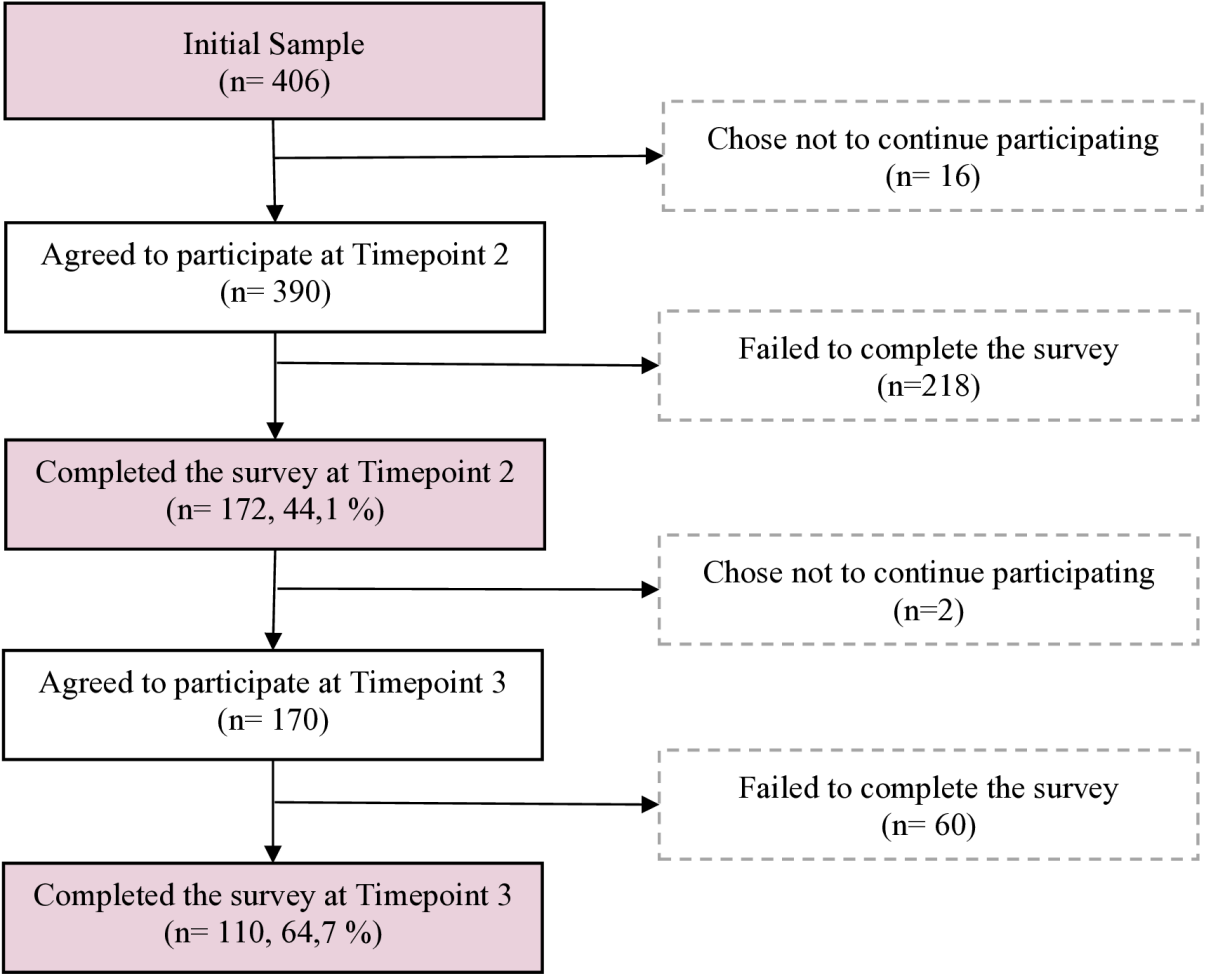
Participant flow diagram.

Out of the initial sample of 406 participants who completed the first time point, 390 agreed to participate in the second time point, and 172 (44.10%) completed the questionnaire. In the third time point, 170 participants expressed willingness to participate, and 110 (64.70%) completed the questionnaire (see Fig 2).

The attrition rate between time points was considered missing data. Little’s MCAR test was performed to assess the missingness pattern, revealing that the missing data were not random (Chi-Square = 23.990, df = 20, p = .243). Therefore, the final sample used in the analysis included data from 110 participants who completed all three-time points. Characteristic of consented eligible participants is presented in Table 1.

Table 1 presents the demographic characteristics of all consented eligible participants across the three-time points of the study. The third column represents participants included in the complete case analysis. This subset consists of individuals who remained in the study until the final time point, providing a basis for analyzing longitudinal trends. Consistent with the gender distribution in the psychology profession in Indonesia (ipkindonesia.or.id), most of the 110 participants were female (n = 104, 94.5%). Participants ranged from 21 to 70 years, averaging 37.48 years (SD = 9.78). Their working experience ranged from 1 to 37 years, averaging 7.44 years (SD = 6.89). Regarding marital status, 76 participants (69.1%) reported being married.

**Fig 3:**
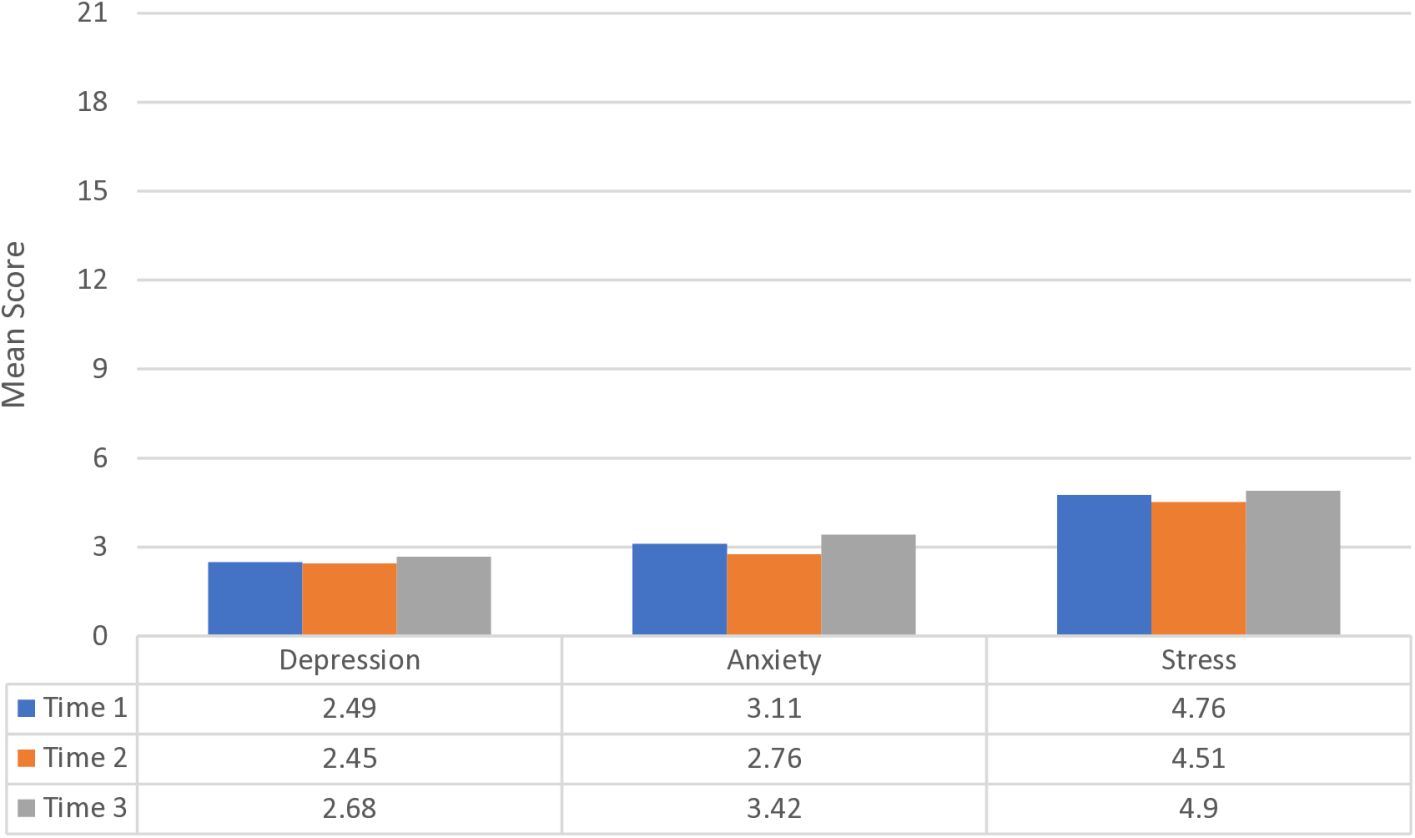
Depressive Symptoms, Anxiety, and Stress in Three Points Measurement.

Fig 3 shows the mean score of depressive symptoms, anxiety, and stress in three points measurement. The Friedman test reveals the absence of statistically significant disparities in the levels of depressive symptoms [χ^2^_F_(2) = 1.46; **p* *> 0.05], anxiety [χ^2^_F_(2) = 1.77; **p* *> 0.05], and stress [χ^2^_F_(2) = 0.93; **p* *> .05].

Table 2 illustrates the distribution of participants categorized into normal, mild, moderate, severe, and extremely severe, according to Lovibond and Lovibond’s percentile cut-offs [44]. The results indicate that the prevalence rates of moderate-to-extremely severe depression symptoms ranged from 10.9% to 14.5%, while the prevalence rates of anxiety symptoms ranged from 22.7% to 30.9%, and the prevalence rates of stress symptoms ranged from 11.8% to 14.5%.

**Table 2:** The Level of DASS-21 Depression, DASS-21 Anxiety, and DASS-21 Stress.

		Timepoint 1	Timepoint 2	Timepoint 3
**Depression**	Moderate to extremely severe	14 (12.7%)	12 (10.9%)	16 (14.5%)
	Normal to mild	96 (87.3%)	98 (89.1. %)	94 (85.5%)
**Anxiety**	Moderate to extremely severe	30 (27.3%)	25 (22.7%)	34 (30.9%)
	Normal to mild	80 (72.7%)	85 (77.3%)	76 (69.1%)
**Stress**	Moderate to extremely severe	13 (11.8%)	13 (11.8%)	16 (14.5%)
	Normal to mild	97 (88.2%)	97 (88.2%)	94 (85.5%)

Table 3 presents the means and standard deviations of the personality traits and self-compassion.

**Table 3:** Descriptive Statistics of Personality Traits and Self-Compassion.

	Mean (SD)	
	W1	W2
**Extraversion**	3.73 (0.55)	–
**Agreeableness**	4.17 (0.46)	–
**Conscientiousness**	3.83 (0.58)	–
**Neuroticism**	2.34 (0.65)	–
**Openness**	3.72 (0.45)	–
**Self-Compassion**	3.92 (0.49)	3.89 (0.48)
**Self-Kindness**	4.01 (0.65)	3.91 (0.60)
**Common Humanity**	4.11 (0.74)	4.06 (0.70)
**Mindfulness**	4.11 (0.62)	4.01 (0.57)
**Self-judgment**	2.27 (0.70)	2.18 (0.69)
**Isolation**	2.18 (0.81)	2.20 (0.86)
**Over Identification**	2.23 (0.74)	2.25 (0.71)

Table 4 presents the hierarchical linear regression analysis predicting depression, anxiety, and stress at Time 3 (T3). For depression (T3). In Model 1, marital status was associated with depression (T3). In Model 2, marital status and openness emerged as predictors of depression (T3). In Model 3, depression (T1) predicts depression (T3), and in Model 4, depression (T1) and depression (T2) predict depression (T3). For anxiety (T3), In Model 2, extraversion and conscientiousness emerged as predictors of anxiety (T3). In Model 3, extraversion, conscientiousness, and anxiety (T1) predict anxiety (T3), and in Model 4, anxiety (T1) and anxiety (T2) predict anxiety (T3). For stress (T3). In Model 1, age was associated with stress (T3). In Model 2, age and conscientiousness emerged as predictors of stress (T3). In Model 3, age, conscientiousness, and stress (T1) predict stress (T3), and in Model 4, conscientiousness and stress (T2) predict stress (T3).

**Table 4:** Results of the hierarchical linear regression analysis predicting depression, anxiety and stress (T3).

	Model 1			Model 2			Model 3			Model 4		
	β	t	p	β	t	p	β	t	p	β	t	p
**Dependent variable: Depression (T3)**												
(Constant)		−0.43	0.67		1.78	0.08		1.31	0.19		0.73	0.47
Gender^a^	0.17	1.90	0.06	0.12	1.36	0.18	0.14	1.86	0.07	0.12	1.74	0.09
Age	−0.23	−1.69	0.09	−0.22	−1.58	0.12	−0.17	−1.51	0.13	−0.10	−0.89	0.37
Marital Status^b^	**0.27**	2.73	0.01	**0.35**	**3.29**	**0.00**	0.16	1.71	0.09	0.10	1.14	0.26
Working Experience	−0.01	−0.10	0.92	0.01	0.04	0.97	0.00	0.02	0.98	−0.06	−0.57	0.57
Extraversion				−0.05	−0.44	0.66	0.00	0.02	0.99	0.07	0.83	0.41
Agreeableness				−0.02	−0.21	0.83	−0.08	−0.88	0.38	−0.10	−1.08	0.28
Conscientiousness				−0.10	−0.98	0.33	−0.05	−0.57	0.57	−0.02	−0.26	0.80
Neuroticism				−0.10	−0.87	0.38	−0.06	−0.66	0.51	−0.03	−0.30	0.77
Openness				**−0.25**	**−2.37**	**0.02**	−0.15	−1.80	0.07	−0.13	−1.53	0.13
Self-Compassion T1							−0.04	−0.39	0.70	0.01	0.11	0.92
Depression T1							**0.49**	**4.12**	**0.00**	**0.38**	**3.10**	**0.00**
Anxiety T1							−0.07	−0.71	0.48	0.02	0.20	0.84
Stress T1							0.15	1.21	0.23	−0.02	−0.12	0.90
Self-Compassion T2										−0.03	−0.31	0.76
Depression T2										**0.33**	**2.85**	**0.01**
Anxiety T2										0.05	0.48	0.63
Stress T2										0.00	−0.03	0.98
Model	Adj. R^2^ = .15			Adj. R^2^ = .19			Adj. R^2^ = .47			Adj. R^2^ = .53		
	F(4, 105) = 5.84			F(9, 100) = 3.74			F(13, 96) = 8.56			F(17, 92) = 8.21		
	p = .00			p = .00			p = .00			p = .00		
Change in R^2^	.18			.07			.29			.07		
	p = .00			p = .11			p = .00			p = .01		
**Dependent variable: Anxiety (T3)**												
(Constant)		0.93	0.35		1.95	0.05		1.40	0.17		1.07	0.29
Gender^a^	0.05	0.50	0.62	−0.01	−0.12	0.91	0.05	0.66	0.51	0.06	0.85	0.40
Age	−0.22	−1.57	0.12	−0.23	−1.67	0.10	−0.14	−1.22	0.22	−0.01	−0.12	0.90
Marital Status^b^	0.19	1.92	0.06	0.17	1.62	0.11	0.08	0.84	0.40	0.13	1.43	0.16
Working Experience	−0.03	−0.20	0.84	−0.05	−0.37	0.71	−0.05	−0.44	0.66	−0.16	−1.53	0.13
Extraversion				**−0.26**	**−2.49**	**0.01**	**−0.24**	**−2.67**	**0.01**	−0.10	−1.13	0.26
Agreeableness				0.22	1.93	0.06	0.09	0.91	0.37	0.03	0.35	0.73
Conscientiousness				**−0.27**	**−2.54**	**0.01**	**−0.20**	**−2.25**	**0.03**	−0.12	−1.41	0.16
Neuroticism				−0.02	−0.20	0.84	−0.05	−0.57	0.57	−0.02	−0.22	0.83
Openness				−0.06	−0.57	0.57	−0.04	−0.41	0.69	−0.11	−1.37	0.17
Self-Compassion T1							0.05	0.52	0.61	0.10	0.93	0.36
Depression T1							0.02	0.17	0.87	−0.01	−0.05	0.96
Anxiety T1							**0.41**	**3.78**	**0.00**	**0.33**	**3.11**	**0.00**
Stress T1							0.20	1.56	0.12	0.02	0.11	0.91
Self-Compassion T2										−0.11	−0.95	0.35
Depression T2										0.01	0.08	0.93
Anxiety T2										**0.45**	**4.21**	**0.00**
Stress T2										−0.04	−0.31	0.76
Model	Adj. R^2^ = .13			Adj. R^2^ = .24			Adj. R^2^ = .51			Adj. R^2^ = .62		
	F(4, 105) = 3.98			F(9, 100) = 3.59			F(13, 96) = 7.54			F(17, 92) = 8.75		
	p = .01			p = .00			p = .00			p = .00		
Change in R^2^	.13			.11			.26			.11		
	p = .01			p = .02			p = .00			p = .00		
**Dependent variable: Stress (T3)**												
(Constant)		2.22	0.03		2.56	0.01		2.65	0.01		1.99	0.05
Gender^a^	−0.04	−0.45	0.65	−0.08	−0.81	0.42	−0.03	−0.43	0.67	−0.01	−0.19	0.85
Age	−0.30	−2.11	0.04	**−0.27**	**−1.95**	**0.05**	**−0.24**	**−2.09**	**0.04**	−0.12	−1.04	0.30
Marital Status^b^	0.15	1.47	0.14	0.17	1.58	0.12	0.00	−0.02	0.98	0.02	0.22	0.83
Working Experience	0.04	0.31	0.76	0.02	0.16	0.88	0.04	0.34	0.74	−0.04	−0.37	0.71
Extraversion				−0.03	−0.24	0.81	−0.07	−0.76	0.45	0.00	−0.04	0.97
Agreeableness				0.10	0.81	0.42	−0.02	−0.19	0.85	−0.02	−0.24	0.81
Conscientiousness				**−0.31**	**−2.89**	**0.00**	**−0.26**	**−2.89**	**0.00**	**−0.19**	**−2.25**	**0.03**
Neuroticism				−0.03	−0.26	0.80	−0.03	−0.30	0.77	0.02	0.21	0.83
Openness				−0.12	−1.11	0.27	−0.04	−0.42	0.68	−0.06	−0.70	0.49
Self-Compassion T1							−0.04	−0.41	0.68	−0.05	−0.42	0.68
Depression T1							0.09	0.72	0.47	0.12	0.93	0.36
Anxiety T1							−0.13	−1.17	0.25	−0.10	−0.92	0.36
Stress T1							**0.57**	**4.37**	**0.00**	0.18	1.13	0.26
Self-Compassion T2										−0.03	−0.25	0.80
Depression T2										−0.03	−0.22	0.83
Anxiety T2										0.12	1.05	0.30
Stress T2										**0.38**	**2.51**	**0.01**
Model	Adj. R^2^ = .13			Adj. R^2^ = .22			Adj. R^2^ = .49			Adj. R^2^ = .57		
	F(4, 105) = 4.03			F(9, 100) = 3.11			F(13, 96) = 7.07			F(17, 92) = 7.27		
	p = .00			p = .00			p = .00			p = .00		
Change in R^2^	.13			.09			.27			.08		
	p = .00			p = .06			p = .00			p = .00		

Abbreviations: T1, Time point 1; T2, Time point 2; T3, Time point 3

* p < 0.05; ** p < 0.01

a Male = 1, Female = 2

b Married = 1, Non Married = 2

legend

In addition, we analyzed a hierarchical model using data from all time points (see S1 File). The results indicated that self-compassion (T1) and (T2) was associated with DASS at the same time points; however, its effect diminished over time and did not extend to (T3). Furthermore, stress emerged as a predictor of both depression and anxiety.

## Discussion

The present study examined the temporal trends of depressive symptoms, anxiety, and stress among Indonesian psychologists during the COVID-19 pandemic. Additionally, we investigated the influence of personality traits and self-compassion on the levels of depressive symptoms, anxiety, and stress experienced by this population. Interestingly, our study did not find significant alterations in depressive symptoms, stress, and anxiety among psychologists during the COVID-19 pandemic 2021. The prevalence of depressive symptoms, anxiety, and stress ranged from 10.9%−14.5%; 22.7%−30.9%; and 11.8%−14.5%; respectively. Depression at T1 and T2 predicts depression at T3. Similarly, the anxiety and stress at T1 and T2 predict the anxiety and stress at T3. Self-compassion at T1 and T2 was associated with depression, anxiety, and stress at the same time points; however, its effect diminished over time and did not extend to T3. Furthermore, stress emerged as a predictor of both depression and anxiety. Regarding sociodemographics, being single predicts depression and younger age predicts stress. In terms of personality traits, lower openness predicts depression, lower extraversion, and lower conscientiousness predicts anxiety, while lower conscientiousness predicts the level of stress.

Our findings align with a study conducted among Brazilian psychologists, which found no changes in depressive symptoms, anxiety, and stress levels between 2020 and 2021 [51]. However, it is important to note that Campos et al. utilized non-paired samples, whereas our study employed the same samples at different time points, allowing for direct comparison among individuals. Our findings contrast with a previous study [52] among Australian hospital clinical staff at two-time points in 2021 and a study investigating depression and anxiety among doctors and nurses healthcare workers in Singapore in 2020 and 2021 [53]. Using the same measurements (DASS-21), both studies demonstrated a significant increase in depressive symptoms, anxiety, and stress over time. Although comparing results is challenging and has several shortcomings, exploring why Indonesian psychologists score lower on these questionnaires is interesting. One possible explanation for this difference could be the timing of our data collection, which occurred during the second year of the pandemic. By this point, individuals may be a bit more accustomed to the ongoing challenges presented by the pandemic. Another potential explanation could be that in 2021, there was a sense of optimism due to the development of vaccines. Another explanation could be that since June 2021, the Indonesian government has implemented the “new normal era.” This means that they were gradually reopening economic and social activities while enforcing health protocols such as physical distancing, mask-wearing, and increased hygiene measures to prevent the spread of the virus. These factors may have instilled a sense of hope and contributed to the observed differences in psychological well-being among Indonesian psychologists compared to the earlier studies conducted during the pandemic’s peak. While such contrasts are noteworthy, they should be interpreted with caution due to the limited sample size and sampling method.

When comparing our results to previous research utilizing the Depression, Anxiety, and Stress Scale (DASS-21) as a reference scale, we consistently observed lower prevalence rates of depressive symptoms, anxiety, and stress in our sample compared to other studies. For instance, a study conducted among psychologists in Brazil reported a high prevalence of at least moderate symptoms of depression (30–40%), anxiety (25–30%), and stress (25–30%) [51]. Similarly, studies among healthcare workers in Australia found that 21.6% were categorized as moderate to extremely severe depressive symptoms, 28.6% were categorized as moderate to extremely severe anxiety, and 28.0% were categorized as moderate to extremely severe stress scores [54]. Another study conducted in Brazil among healthcare workers reported moderate to extremely severe symptoms of depression, anxiety, and stress in 48.6%, 55.0%, and 47.9% of the participants, respectively [55]. Compared to Indonesian populations, a study among medical students in Indonesia using the DASS-21 revealed that 22.2% reported symptoms of depression, and 48.1% reported anxiety [56]. A study on the general population found that 32.45–37.22%, 44.11–46.42%, and 37.01–49.66% of respondents exhibited signs of psychological symptoms according to anxiety, depression, and stress scores [57]. These comparisons highlight the relatively lower prevalence of depressive symptoms, anxiety, and stress in our sample compared to the studies conducted among different populations and in various countries. Our study revealed that depression, anxiety, and stress in T1 & T2 predict depression, anxiety, and stress in T3. This discovery corroborates the widely acknowledged phenomenon that prior experiences of depression serve as the most reliable predictor for future occurrences [58].

The transient association between self-compassion and depression, stress, and anxiety observed in our study aligns with findings from a longitudinal study [59], which explored the role of self-compassion over time in relation to perceived stress, depression, and anxiety among college students. Their study suggested that while high self-compassion may promote faster recovery from stress, its long-term impact may diminish as new stressors and life experiences increasingly shape emotional well-being. A similar pattern may be explained by elevated levels of compassion fatigue, particularly in healthcare workers, whose continuous role in providing care and compassion to others can gradually lead to emotional exhaustion [60]. In this context, compassionate efforts may be primarily directed toward others, leaving limited resources for self-compassion. This interpretation is consistent with findings by Matos et al. [61], who reported a decline in self-compassion among healthcare practitioners between the second and third measurement time points.

Our study found that stress emerged as a significant predictor of both depression and anxiety. This finding aligns with previous research, among healthcare workers [62] and among nurses [63] during the COVID-19 pandemic, both of which demonstrated a strong link between stress and mental health outcomes. One possible explanation for this relationship is the Cognitive-Emotional Downward Spiral [64]. This mechanism suggests that stress can trigger negative emotional responses, which in turn amplify the perception of stress, creating a self-reinforcing cycle that exacerbates both anxiety and depression.

Our study identified an association between being unmarried and higher levels of depressive symptoms. These findings align with [65,66], which reported that being single is a risk factor for adverse mental health outcomes. Another study identified that single status predicted 34% of depressive symptoms [65]. Their findings suggest that individuals who are not in a relationship may experience higher levels of depressive symptoms than those in a relationship. Similarly, a study investigated the relationship between marital status and mental health and found that singles exhibited higher levels of depression, anxiety, and stress than married individuals [66]. This finding suggests that being single may contribute to increased psychological distress. Several factors may influence this finding. The impact of social isolation on psychological distress during the pandemic has been widely recognized [67]. Single individuals may encounter challenges in establishing and maintaining social connections and support systems, particularly during lockdowns and restricted social interactions. This lack of social connection can give rise to feelings of loneliness and detachment, both of which have been linked to elevated levels of depressive symptoms and anxiety [68]. Additionally, the limited availability of physical touch during the pandemic can further exacerbate feelings of isolation and emotional distress, especially for single individuals who may not have a partner to provide support and companionship [69]. These factors collectively contribute to the higher psychological burden experienced by single individuals during these challenging times.

Our study revealed that younger age predicts a higher level of stress. Similar to other COVID-19 research, age is related to stress during COVID-19 [70]. One potential explanation might be that younger individuals are more attuned to the adverse economic implications and future employment prospects resulting from the pandemic [71]. Furthermore, the prevalence of frustrating COVID-19 news coverage, particularly on social media platforms, may disproportionately impact the mental well-being of younger generations [72].

Our research indicates that certain personality traits are predictive of mental health issues. Specifically, a lower level of openness is associated with higher levels of depression, a relationship supported by prior research [29,73]. Individuals with a higher degree of openness tend to exhibit curiosity in exploring various coping mechanisms and demonstrate creativity in adapting these strategies flexibly [74]. Additionally, lower levels of extraversion and conscientiousness are linked to elevated anxiety levels. This finding is consistent with previous studies [75,76]. Furthermore, lower conscientiousness predicts higher stress levels, aligning with a previous longitudinal study among US citizens [77] and undergraduate students in Turkey [75].

Our study has several strengths. Firstly, it stands as one of the pioneering investigations to report the prevalence and associated factors of depressive symptoms, anxiety, and stress among psychologists in Indonesia during the pandemic. We only found one article that reported the prevalence of depression, anxiety, and stress symptoms among Brazilian psychologists [51]. Additionally, a longitudinal design enabled the exploration of temporal trends, offering valuable insights into potential causal pathways underlying mental health issues within the psychologist population. This study highlights that determining important protective factors to maintain good mental health for psychologists is important. Not only for future periods of societal stress, but also in general, to know more about what helps psychologists in Indonesia to stay healthy. Factors such as being married, higher openness, and extraversion may thus be protective factors. Future interventions that target mental health in mental health care professionals, and psychologists specifically, can benefit from this knowledge, and when in situations of immense stress (such as a pandemic), interventions should be targeted at psychologists who may be more vulnerable to mental health symptoms.

We also acknowledge several important limitations of this study. First, regarding generalizability and sampling, our findings may be confined to the specific group of psychologists who participated and may not be fully applicable to the wider population of psychologists in Indonesia or to psychologists in other countries. Recruitment relied on social media platforms and professional networks, which may have introduced self-selection bias. This approach likely favored individuals who were more active online or more interested in the topic. In addition, because the research team was based in Java, the most digitally connected island in Indonesia, psychologists from urban centers may have been overrepresented, while those working in rural or remote regions with limited internet access may have been underrepresented. As a result, the sample may not fully capture the diversity of psychologists across Indonesia, consistent with concerns raised in prior research on digital recruitment [78].

Second, with respect to measurement and methodology, our study relied on self-report surveys. These measures, while efficient for large-scale data collection, are vulnerable to response bias and have inherent limitations in providing comprehensive clinical assessments. They primarily capture symptom severity rather than supporting diagnostic classification according to DSM-5 criteria. Additionally, missing data were addressed using complete-case analysis. Although this method is commonly applied, it can introduce bias in longitudinal designs if data are not missing completely at random [79]. Given the small proportion of missing data, we considered this approach acceptable; however, alternative techniques such as multiple imputation or full information maximum likelihood could provide less biased estimates [80].

Third, regarding attrition and retention, we lacked detailed information on why some participants dropped out across time points. The use of online data collection may have contributed to this pattern, as participants who remained engaged were likely those who were more motivated or emotionally stable. This selective retention mirrors findings from a longitudinal study in Indonesia during the COVID-19 pandemic Jaya et al. [81]. It is also unclear whether incentives influenced retention, as evidence from the UK suggests that incentives may improve participation but are not necessarily the primary driver, with other factors—such as communication quality, perceived importance of the study, and participation flexibility—playing a significant role [82]. In the pandemic context, health concerns and shifting priorities may have further complicated retention.

Finally, in terms of sample size and statistical power, the final sample fell slightly below the estimated requirement from G*Power. Although the shortfall was modest and regression analyses still had adequate power, this may have reduced sensitivity to detect smaller effects. Consequently, non-significant results should be interpreted with caution.

## Conclusion

In conclusion, this study represents the first investigation into the temporal trend of mental health among psychologists in Indonesia during the COVID-19 pandemic. The findings indicate a relatively stable and low prevalence of depressive symptoms, anxiety, and stress over the second year of the pandemic. Furthermore, being married, older age, higher openness to experience, higher extraversion, and higher conscientiousness emerged as potential protective factors against mental health issues. At the same time, stress emerged as a predictor of both depression and anxiety. This finding underscores the importance of recognizing stress not only as a theoretical predictor but also as a practical target for intervention. In workplace contexts, stress-management programs, peer support networks, and organizational policies that reduce workload pressures could help mitigate its negative impact on psychologists’ well-being.

To further advance the field, future research should consider utilizing larger and more diverse samples, employing a combination of self-report measures and clinical assessments for comprehensive diagnostic evaluation. Moreover, exploring the generalizability of findings to psychologists in different cultural contexts would contribute to a more comprehensive understanding of the mental health of psychologists during very stressful times, such as a pandemic. The capability of Indonesian psychologists to effectively manage the psychological stress associated with the COVID-19 pandemic is perceived as powerful. However, further research is warranted to investigate additional protective factors and explore why Indonesian psychologists are less prone to psychiatric symptoms than other populations. In addition, it would be interesting to study further sociodemographic characteristics and related factors as much as possible to give a wider view and understanding of this study, such as the number of working hours, the type of counseling, the age of clients, and work stress or work burnout.

## Supporting information

S1 File Correlation and Hierarchical Regression All Waves.xlsx. This file contains the full correlation matrix and hierarchical regression analyses for all study waves, including predictor and outcome variables, standardized coefficients, and significance levels.
(XLSX)
